# Information at Work: Information Management in the Workplace

**DOI:** 10.5195/jmla.2020.905

**Published:** 2020-04-01

**Authors:** David Farris

**Affiliations:** Research Medical Library, University of Texas MD Anderson Cancer Center, Houston, TX, dpfarris@mdanderson.org, https://orcid.org/0000-0002-9224-9270

Work is an important aspect of life: the types of work people do provide them with a sense of identity and purpose. Work also places people in distinct environments that require them to interact with information to accomplish tasks specific to their professions and workplaces. Although there has been a great deal of research into the use, organization, and management of information in the information science field, investigations into these topics in the framework of the workplace have generally taken place in other academic disciplines that are closely related to information science. Thus, the editors of *Information at Work: Information Management in the Workplace* state that their aim “is to present the full spectrum of workplace information research...from an information science perspective” (p. 148).

This deceptively slim volume is organized into seven chapters with a foreword by Annemaree Lloyd, a noted scholar of information literacy in the context of workplaces and communities of learning. The introductory and concluding chapters are written by the editors, and the remaining chapters are authored by contributors who specialize in the topic of that chapter. The first chapter provides an updated understanding of the inherent complexities of the workplace and work life in the twenty-first century by examining established information science research about the workplace. The final chapter summarizes the research and perspectives from the preceding chapters and offers an updated version of Robert S. Taylor’s “Information Use Environment” (IUE) model. The intermediate chapters examine workplace information from myriad contexts: (1) the activities and tasks performed in the workplace, (2) the information culture of an organization, (3) the individual and collective management of information, (4) the information objects that workers use, and (5) the characteristics of workplace information objects.

*Information at Work: Information Management in the Workplace* provides a current assessment of workplace information management in the field of information science and uses Taylor’s scholarship and IUE model as a recurring motif to compare and frame the discussions throughout the book. Building on the research of Taylor and other experts in the information science field reveals the intense thought and analysis the authors have undertaken to present their findings.

There are no particularly unique features included in the book; it does not include a glossary because each chapter defines the concepts it examines. However, the index is more than adequate and provides an exhaustive list of terms and cross-references for locating information in the text. Statements and ideas in each chapter are supported with in-text citations to other scholarly works, and a comprehensive reference list follows every chapter, which not only gives the authors’ findings and conclusions greater credibility, but also enables the user to further research the topic. Several chapters include figures that illustrate various concepts such as the relationship between one’s role at work and the processes used to accomplish work as well as the components of information culture that affect information practices.

Due to the theoretical nature of this book, it is highly recommended to be added to academic library collections, especially those supporting a university with a library and information science program. Scholars, researchers, and library and information science graduate students would all benefit from reading this book in its entirety or selected chapters of interest. All of the editors and contributors are faculty members and scholars at European universities and affiliates of the European Network for Workplace Information, a network of information professionals from academia and the private sector that aims to improve work satisfaction through research. Each author is a respected scholar in his or her specialty and has published widely. According to Scopus, the group of authors collectively has over 400 publications and an average *h*-index score of 11.

Information professionals who are employed at medical and health sciences libraries may also be interested in *Information at Work*, because the book uses five fictional personas to illustrate how the various concepts explained throughout the text are relevant to employees in different occupations and work environments, including a cardiologist at a large urban hospital. Due to the dynamic and unpredictable nature of the health care workplace, using a medical professional to help illustrate the concepts provides insight into the workplace information needs that medical librarians can fill.

***David Farris, AHIP****, dpfarris@mdanderson.org, https://orcid.org/0000-0002-9224-9270, Research Medical Library, University of Texas MD Anderson Cancer Center, Houston, TX*

